# Genotype classification of *Moraxella bovis* using MALDI-TOF MS profiles

**DOI:** 10.3389/fmicb.2022.1057621

**Published:** 2022-12-08

**Authors:** Hannah G. Olson, John Dustin Loy, Michael L. Clawson, Emily L. Wynn, Matthew M. Hille

**Affiliations:** ^1^School of Veterinary Medicine and Biomedical Sciences, Institute for Agriculture and Natural Resources, University of Nebraska-Lincoln, Lincoln, NE, United States; ^2^United States Department of Agricultural, Agricultural Research Service, United States Meat Animal Research Center, Clay Center, NE, United States

**Keywords:** *Moraxella bovis*, MALDI-TOF MS, infectious bovine keratoconjunctivitis, bovine pinkeye, genotype

## Abstract

*Moraxella bovis* (*M. bovis*) is regarded as a causative agent of infectious bovine keratoconjunctivitis (IBK), the most common ocular disease of cattle. Recently, whole genome sequencing identified the presence of two distinct genotypes within *M. bovis* that differ in chromosome content, potential virulence factors, as well as prophage and plasmid profiles. It is unclear if the genotypes equally associate with IBK or if one is more likely to be isolated from IBK lesions. We utilized 39 strains of *M. bovis* that had previously undergone whole genome sequencing and genotype classification to determine the utility of matrix-assisted laser desorption/ionization time-of-flight mass spectrometry (MALDI-TOF) to accurately genotype *M. bovis* strains. We successfully developed two biomarker models that accurately classified strains according to genotype with an overall accuracy of 85.8–100% depending upon the model and sample preparation method used. These models provide a practical tool to enable studies of genotype associations with disease, allow for epidemiological studies at the sub-species level, and can be used to enhance disease prevention strategies.

## Introduction

Infectious bovine keratoconjunctivitis (IBK) is the most common ocular disease of cattle (Brown et al., 1998; Kneipp, 2021). IBK clinically presents as a herd-level disease that is often seasonal and can occur with high morbidity (Kneipp, 2021). Clinical signs of IBK include corneal ulceration, lacrimation, conjunctivitis, blepharospasm and potential blindness in severe cases (Kneipp, 2021). *Moraxella bovis* (*M. bovis*) is regarded as the most strongly associated causal agent of IBK, as the disease can be reproduced experimentally in calves by inoculating the cornea with *M. bovis* (Rogers et al., 1987; Beard and Moore, 1994). Other infectious agents such as *Moraxella bovoculi* (*M. bovoculi*), *Mycoplasma bovoculi,* and bovine herpesvirus – type 1 are often recovered from lesions or are found associated with ocular disease, but thus far experimental inoculation of calves with these agents has not produced clinical signs consistent with IBK (Rosenbusch and Ostle, 1986; George et al., 1988; Angelos et al., 2007; Angelos, 2010). Additional environmental factors such as face flies, ultraviolet light, dusty conditions, and tall grasses are also thought to play a role in IBK development (Hughes et al., 1965, 1968; Gerhardt et al., 1982; Hall, 1984; Kopecky et al., 1986; Smith, 2012; Maier et al., 2021a). A precise and current estimate of the economic cost of IBK is lacking, but previous studies have estimated the impact to be between $150–$226 million dollars in the United States alone (Killinger et al., 1977; Hansen, 2001; Dennis and Kneipp, 2021). The economic losses associated with IBK are due to the costs of treatment as well as decreased average daily gain in affected calves (Thrift and Overfield, 1974).

Prevention of IBK is often focused on vaccination and minimizing fly load (Sheedy et al., 2021). There are a number of fully licensed *M. bovis* vaccines, a single conditionally approved *M. bovoculi* vaccine, and autogenous vaccine formulations available from different manufacturers in the United States. Under experimental fields conditions, these vaccine formulations have all had mixed results in terms of preventing IBK regardless of the formulation, route of administration, or antigen makeup (Smith et al., 1990; Davidson and Stokka, 2003; O'Connor et al., 2011; Cullen et al., 2017; Hille et al., 2022).

In the United States, tetracycline and tulathromycin are the only antibiotics with label indications for IBK whereas florfenicol also has a label indication for IBK in Canada (Bio-Mycin 200 (oxytetracline) [package insert]. Duluth, GA; Boehringer Ingelheim Animal Health United States Inc. 2019, Draxxin (tulathromycin) [package insert] Kalamazoo, MI: Zoetis Inc. 2018, Nuflor (florfenicol) [package insert] Kirkland, Quebec, Canada, Merck Animal Health Intervet Canada Corp. 2019). The use of eye patches as an aid in treatment was recently shown to promote healing of corneal ulcers associated with IBK (Maier et al., 2021b).

A secreted repeats-in-toxin (RTX) exotoxin and a type IV pilus protein are the two main virulence factors possessed by *M. bovis* required for IBK development (Jayappa and Lehr, 1986; Clinkenbeard and Thiessen, 1991; Ruehl et al., 1993; Beard and Moore, 1994). Recently, two distinct genotypes of *M. bovis* were characterized that shared a core of 2,015 genes with an additional 121 genes specific to genotype 1 and 186 genes specific to genotype 2 (Wynn et al., 2022). The genotypes possess different sequence variants of RTX and different plasmid profiles. Specifically, only one genotype possessed plasmids containing filamentous hemagglutinin, a known virulence factor in other pathogens. These differences suggest the two genotypes may not be equally associated with IBK although this has not been proven.

Matrix-assisted laser desorption/ionization time-of-flight mass spectrometry (MALDI-TOF MS) is a commonly used method in diagnostic and research laboratories for the identification of bacteria (Seng et al., 2009; Clark et al., 2013; Sandalakis et al., 2017). Within *Moraxella* spp., MALDI-TOF MS has previously been used to accurately distinguish between *M. bovis* and *M. bovoculi* species, as well as distinguish genotypes within *M. bovoculi* (Robbins et al., 2018; Hille et al., 2020). Given the recent characterization of genotypes 1 and 2 of *M. bovis*, we hypothesized that MALDI-TOF MS may provide a timely and accurate method of genotype classification within this species as well. A rapid method to characterize strains of *M. bovis* according to genotype would allow for classification of a large number of disease associated strains to determine potential associations with disease in real-time as part of the bacterial identification process.

## Materials and methods

### Bacterial strains and culture conditions

Thirty seven of the 39 strains of *M. bovis* used for this study had previously undergone bacterial identification, whole genome sequencing and assembly into closed, circular chromosomes, and were classified according to genotype (Wynn et al., 2022). An additional two strains were genotyped from Illumina libraries using two methods: First, Illumina library fastq files were converted to fasta BLAST databases (Camacho et al., 2009) and previously identified genotype 1 and 2 specific genes (Wynn et al., 2022) were used as BLAST queries to find genotype specific gene sequence in the Illumina libraries. The strains consisted of ten genotype 1 and 29 genotype 2 strains. The strains primarily originated from diagnostic case submission samples from cattle with IBK that were submitted to the Nebraska Veterinary Diagnostic Center. The state of origin for each strain is shown in Table 1.

**Table 1:** State of origin and model groups assigned to the 39 strains used in this study.

Genotype	Model Group	Location
1	Generation	Nebraska (4) Florida (1)
1	Validation	Nebraska (2) Indiana (1) Saskatchewan, Canada (1) Wisconsin (1)
2	Generation	Kansas (4) Oregon (1) Minnesota (1) Nebraska (2) North Carolina (1) West Virginia (1) Montana (1) South Dakota (1) Iowa (1) Saskatchewan, Canada (1) Pennsylvania (1)
2	Validation	Nebraska (2) Kansas (1) Iowa (2) California (1) Texas (1) Illinois (1) Wisconsin (1) Florida (1) Illinois (1) Oregon (1) Oklahoma (1) West Virginia (1

The model group assignment was the same for developing both the GA and QC biomarker models.

Frozen stocks of the strains were plated onto tryptic soy agar (TSA) with 5% sheep blood (Remel, Lenexa, KS) and incubated at 37°C for 24 h in 5% CO_2_. The strains were then passed onto fresh blood agar plates incubated for another 24–48 h in the same conditions, and then pure colony growth was subjected to analysis by MALDI-TOF MS.

### MALDI-TOF MS

MALDI TOF MS spectra was obtained for each of the strains per the manufacturer’s recommendations using two methods, the smear method and the extraction method (Khot et al., 2012). To perform the smear method, a single colony was transferred onto the steel target plate using a wooden applicator and allowed to air dry before applying 1 μl of α-cyano-4-hydroxycinnamic acid (Bruker Daltonics, Billerica, MA). The wells were allowed to dry, and crystallization occurred. Analysis involved using MALDI Bioytyper system (Bruker Daltonik) in a positive linear mode with a mass range of 2–20 kDa m/z with laser frequency of 60 Hz and calibration using a Bacterial Test Standard (Bruker Daltonik). The first ion source had a voltage of 20,000 kV, and the second had a voltage of 18.10 kV with an additional lens voltage of 6.05 kV and a pulsed extraction time of 170 ns.

The extraction method involved using 2–3 colonies from solid media that were incubated for 24–48 h in 5% CO_2_ at 37°C. After incubation, 300 μl of HPLC grade water and the colonies were vortexed until a homogenous mixture formed. Next, 900 μl of 100% ethanol was added and then centrifuged for 2 min at 16,000 xg. The supernatant was removed, and the pellet was allowed to air dry. Then, 25 μl of 70% formic acid and 25 μl of acetonitrile were combined with the pellet and centrifuged as mentioned above. Next, 1 μl of the supernatant was placed onto a well and allowed to air dry. The same 1 μl of matrix solution (α-cyano-4-hydroxycinnamic acid) was then added to each well before MALDI-TOF was performed. For each strain, eight wells were prepared using the extraction method, and each well was analyzed three times resulting in a total of 24 spectra. For the smear method, three wells were prepared and analyzed three times for a total of nine spectra. The spectra profiles were examined, and flat or inconsistent spectra were removed from the analysis.

### Model generation and accuracy

Strains from each genotype were randomly assigned to either a biomarker model generation group or validation group (Table 1). ClinProTools 3.0 software (Bruker Daltonik) was used to develop a biomarker model from the known genotypes within the model generation groups. Two classification algorithms were used to develop the models including genetic algorithm (GA) and quick classifier (QC). After the biomarker models were obtained, their accuracy was manually calculated according to the resulting classifications for each of the strains within the validation groups. The models were developed using spectra obtained via the extraction sample preparation method, and the accuracy of the models was assessed using spectra from both the extraction and smear sample preparation method. Any spectrum classified as “Null Spectrum” by the models was excluded from accuracy calculations. A two sample t-test assuming unequal variance was used to compare the classification accuracy between sample preparation methods and a two sample t-test assuming equal variance was used to compare classification accuracy between the models. The significance of genotype discrimination of individual peaks was determined using the “peak statistic” function within ClinProTools 3.0 after loading all spectra from each genotype.

A main spectrum profile (MSP) was created for each strain using 24 spectra from eight technical replicates using MBT Compass Explorer software (Bruker Daltonik). The MSP peak list function was used to determine the presence or absence of peaks included in the biomarker model for each strain. When present, the magnitude of each peak used in the biomarker model was recorded.

## Results

The peaks included in the biomarker models for this study and the classification accuracies are summarized in Table 2. The GA biomarker model included five peaks and correctly classified 100% (110/110) and 100% (307/307) of validation group spectra obtained using the extraction method for genotype 1 and 2 strains, respectively. Therefore, the overall classification accuracy for extraction method spectra was 100% (417/417). When the smear method spectra were classified, the GA model correctly classified 81.3% (26/32) and 99.8% (122/123) of the genotype 1 and 2 validation spectra, respectively, for an overall accuracy of 95.5% (146/155) which was significantly lower than the extraction method accuracy (*p* = < 0.05).

**Table 2:** Biomarker model characteristics and accuracy results for both the GA and QC biomarker models.

Model	GA	QC
Peaks used (m/z)	6,839	6,854
	6,854	
	7,301	
	8,769	
	9,103	
**Extraction method**		
Recognition Capability	100%	98.56%
Cross Validation	99.79%	98.21%
Classification genotype 1	100% (110/110)	100% (110/110)
Classification genotype 2	100% (307/307)	97.4% (299/307)
**Smear method**		
Classification genotype 1	81.3% (26/32)	100% (32/32)
Classification genotype 2	99.8% (122/123)	82.1% (101/123)

**Figure 1:**
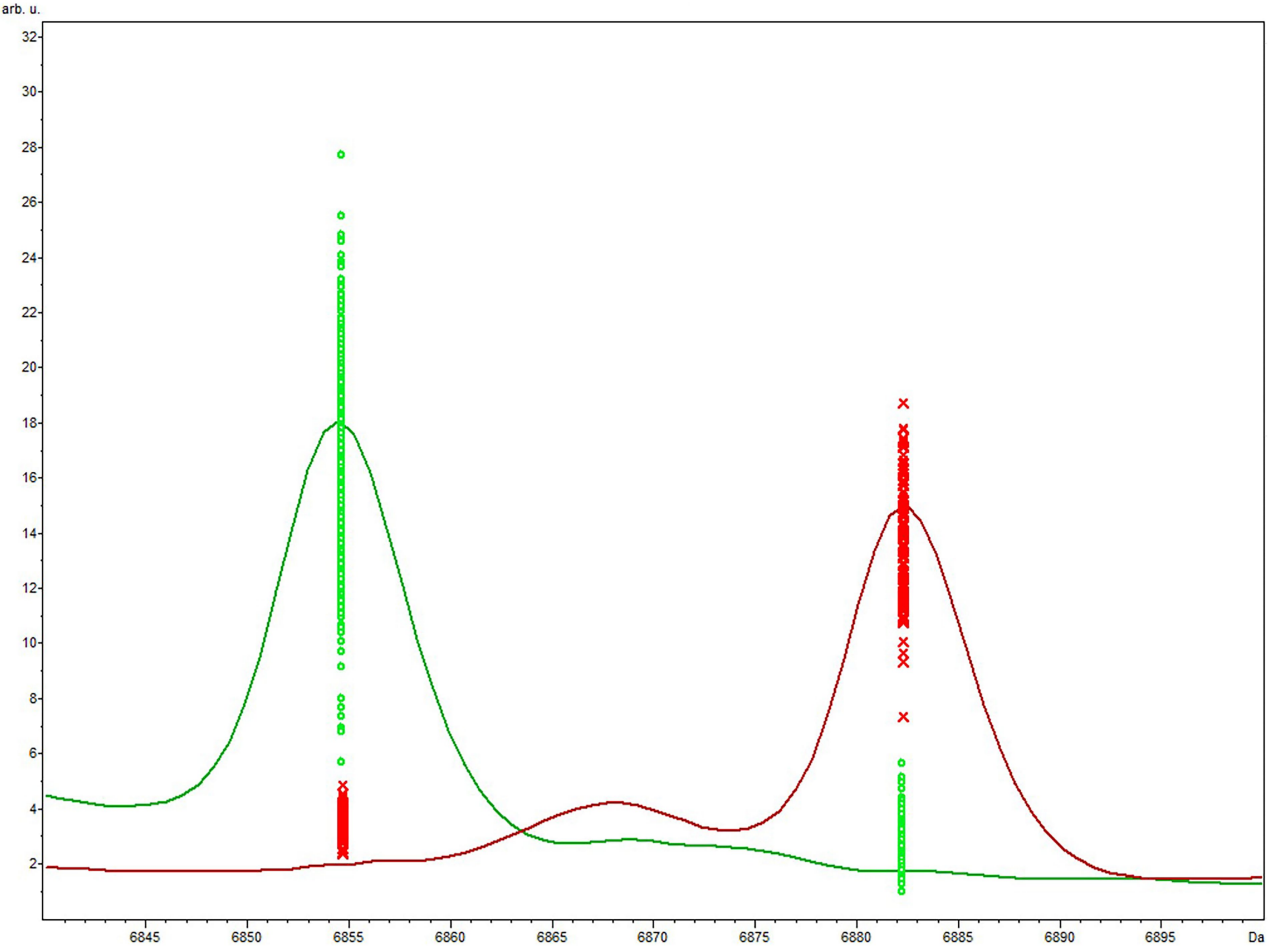
Average genotype 1 (red) vs. genotype 2 (green) spectra focused on the area of peak 6,854 m/z included in the QC biomarker model. The differential expression and magnitude of peaks allows for differentiation of the respective genotypes.

The QC biomarker model incorporated a single peak at 6854 m/z and correctly classified 100% (110/110) and 97.4% (299/307) of the genotype 1 and 2 extraction method validation spectra, respectively. The resulting overall classification accuracy for the extraction spectra was 98.1% (409/417). When classifying spectra obtained using the smear method, the QC model correctly classified 100% (32/32) and 82.1% (101/123) of genotype 1 and 2 spectra, respectively. The overall accuracy for smear spectra was therefore 85.8% (133/155) which was significantly lower than the extraction method accuracy (*p* = < 0.05). The accuracy of the GA model was statistically superior to the QC model using both the extraction method (*p* = < 0.05) and the smear method (*p* = < 0.05) of sample preparation. The highest weighted peak in the GA model was peak 6,854 m/z, which is the same peak used in the QC model. While the GA model showed superior accuracy, the QC model is still highly accurate and is appealing from a practical standpoint since it only uses a single peak. Highlighting the discriminatory power of this single peak would be especially useful if spectra were to be manually evaluated instead of using the ClinProTools 3.0 software. For this reason, we chose to focus on peak 6,854 m/z to examine the presence or absence, as well as the relative magnitude in the MSP profiles of all strains. Peak 6,854 m/z was highly significant between the two genotypes (*p* = < 0.000001) according to the Peak Statistic function within ClinProTools 3.0. This peak was present in all 14 genotype 2 validation MSPs with an average intensity of 24.16 arbitrary units (a.u.) and only present in 1/5 genotype 1 MSPs, and with a substantially lower intensity of only 3.01 a.u. (Table 3). Figure 1 displays the average spectra from each genotype at 6854 m/z as well as the distribution of each individual spectra.

**Table 3:** The presence and intensity of peak 6,854 m/z used in the QC model for each of the validation group strains.

Peak (m/z)	6,854 (Range 6844.26–6864.35)	Average intensity (arbitrary units)
Extraction Method		
Genotype 1	1/5	3.01
Genotype 2	14/14	24.16
Smear Method		
Genotype 1	0/5	0.0
Genotype 2	14/14	38.38

## Discussion

We successfully developed two MALDI-TOF MS biomarker models that accurately classified strains of *M. bovis* according to genotype. The GA model was significantly more accurate than the QC model using both the extraction and smear method. The accuracy of the smear method was significantly less than the extraction method for both models. However, the accuracy of the models using the smear method (95.5% for GA, 85.8% for QC) is likely sufficient given the ease of sample preparation compared to the extraction method. Regardless of the sample preparation method used, replication should be included to increase the discriminatory resolution of peak 6,854 m/z to account for individual profile variation of this peak, particularly when using the smear method. When the MSP of the strains were examined, determining the presence or absence of peak 6,854 m/z, in conjunction with the intensity, was sufficient to differentiate between the two genotypes. Therefore, manual observation of the MSP or individual spectrum profile of an unknown strain for a peak at 6854 m/z can allow for accurate genotype determination without the need for the biomarker model, if ClinProTools 3.0 software is not available.

One limitation for this study is that the collection of *M. bovis* strains with known genotypes is limited since they have only been recently described, and further study of the application of this model to strains more diverse in space and time is warranted. As there is not a standard number of strains or spectra required to generate MALDI-TOF biomarker models, we included 24 spectra from each strain in the model generation portion of this study to capture variability both between and within individual strain spectra. The classification accuracy of the models indicates consistent differences between the genotypes and indicates that these models are a valuable tool to genotype uncharacterized strains with a high degree of accuracy.

MALDI-TOF delivers several benefits over both whole genome sequencing and PCR. While the initial investment in MALDI-TOF capabilities is substantial, the reagents are fewer and costs associated with testing an individual strain is more cost effective and results are available more quickly. Additionally, MALDI-TOF has proven more accurate in identifying members of the genus *Moraxella* to the species level when compared to PCR (Robbins et al., 2018). Additionally, the raw data generated from the instrument as part of the species identification run can be used directly to identify genotypes, providing added value to existing data.

Additional work to determine the relative abundance of each genotype within healthy and IBK affected eyes using MALDI-TOF MS profiles will allow us to determine if either of the genotypes is more likely to be associated with disease. If it is determined that a specific genotype is more likely to be disease-associated, it may be beneficial to preferentially include such strains in future vaccine formulations, particularly for autogenous vaccines. Beyond disease association, the *M. bovis* genotyping models will help determine any geographic or seasonal differences in the abundance of the genotypes as well. Any differences determined in either geographic or seasonal distribution of the genotypes may provide another method for vaccine formulation customization. If both genotypes are represented equally among diseased eyes, the genotype classification will still prove useful in any efforts to decipher any potential differences in the mechanics of pathogenesis and/or the utilization of certain virulence factors. The.XML files for both the GA and QC models developed in this study are available upon request by contacting the corresponding author.

## Data availability statement

The datasets presented in this study can be found in online repositories. The names of the repository/repositories and accession number(s) can be found in the article/supplementary material.

## Author contributions

MH and HO conceptualized the study, performed MALDI-TOF analysis, developed the biomarker models, wrote the first draft, and edited the manuscript. JL conceptualized the study and edited the manuscript. MC and EW genotyped the isolates and edited the manuscript. All authors contributed to the article and approved the submitted version.

## Funding

Funding for this study was provided by the Nebraska Experiment Station with funds from the Animal Health and Disease Research (section 1433) capacity funding program (accession 1017646) through the USDA National Institute of Food and Agriculture. The use of product and company names is necessary to accurately report the methods and results; however, the United States Department of Agriculture (USDA) neither guarantees nor warrants the standard of the products, and the use of names by the USDA implies no approval of the product to the exclusion of others that may also be suitable. The USDA is an equal opportunity provider and employer.

## Conflict of interest

The authors declare that the research was conducted in the absence of any commercial or financial relationships that could be construed as a potential conflict of interest.

## Publisher’s note

All claims expressed in this article are solely those of the authors and do not necessarily represent those of their affiliated organizations, or those of the publisher, the editors and the reviewers. Any product that may be evaluated in this article, or claim that may be made by its manufacturer, is not guaranteed or endorsed by the publisher.
